# Tuneable Giant Magnetocaloric Effect in (Mn,Fe)_2_(P,Si) Materials by Co-B and Ni-B Co-Doping

**DOI:** 10.3390/ma10010014

**Published:** 2016-12-27

**Authors:** Nguyen Van Thang, Niels Harmen van Dijk, Ekkes Brück

**Affiliations:** Fundamental Aspects of Materials and Energy, Department of Radiation Science and Technology, Delft University of Technology, Mekelweg 15, Delft 2629 JB, The Netherlands; N.H.vanDijk@tudelft.nl (N.H.v.D.); E.H.Bruck@tudelft.nl (E.B.)

**Keywords:** magnetic refrigeration, magnetocaloric effect, Fe_2_P, Co substitution, Ni substitution

## Abstract

The influence of Co (Ni) and B co-doping on the structural, magnetic and magnetocaloric properties of (Mn,Fe)${}_{2}$(P,Si) compounds is investigated by X-ray diffraction (XRD), differential scanning calorimetry, magnetic and direct temperature change measurements. It is found that Co (Ni) and B co-doping is an effective approach to tune both the Curie temperature and the thermal hysteresis of (Mn,Fe)${}_{2}$(P,Si) materials without losing either the giant magnetocaloric effect or the positive effect of the B substitution on the mechanical stability. An increase in B concentration leads to a rapid decrease in thermal hysteresis, while an increase in the Co or Ni concentration hardly changes the thermal hysteresis of the (Mn,Fe)${}_{2}$(P,Si) compounds. However, the Curie temperature decreases slowly as a function of the Co or Ni content, while it increases dramatically for increasing B concentration. Hence, the co-substitution of Fe and P by Co (Ni) and B, respectively, offers a new control parameter to adjust the Curie temperature and reduce the thermal hysteresis of the (Mn,Fe)${}_{2}$(P,Si) materials.

## 1. Introduction

The magnetocaloric effect (MCE), which was first described in 1917 by Weiss and Piccard [1,2], corresponds to the change in temperature when a magnetic field is changed under adiabatic conditions or the change in entropy when the field is changed under isothermal conditions. From a thermodynamic point of view, the isothermal magnetic entropy change $\Delta S_{m}$ and the adiabatic temperature change $\Delta T_{ad}$ are two characteristic parameters to evaluate the MCE of a magnetic material. $\Delta S_{m}$ is a measure of how much heat can be transported (at a given temperature) by magnetic means, while $\Delta T_{ad}$ is a measure of how big the temperature difference is that can be achieved in the transfer of the heat to and from the heat transfer fluid [3]. In other words, $\Delta S_{m}$ determines the cooling capacity, and $\Delta T_{ad}$ is directly associated with the driving force of heat transfer and thus determines the cycle frequency. Hence, to evaluate the MCE adequately, both $\Delta S_{m}$ and $\Delta T_{ad}$ need to be taken into account.

Magnetic materials that show a giant MCE have drawn widespread attention in the recent past due to their potential applications for room-temperature magnetic refrigeration [3,4]. Compared to the conventional vapour-compression refrigeration, this cooling technology promises a 25% higher energy efficiency and does not use dangerous and environmentally unfriendly refrigerants such as ozone depleting chemicals (e.g., chlorofluorocarbons (CFCs)), hazardous chemicals (e.g., ammonia (NH${}_{3}$)) or greenhouse gases (e.g., hydrofluorocarbons (HFCs) and hydrochlorofluorocarbons (HCFCs)) [3,5,6]. This makes magnetic refrigeration one of the most promising technologies to replace vapour-compression refrigeration in the near future.

Materials displaying a first-order magnetic transition (FOMT) near room temperature are promising candidates for magnetic refrigeration because these materials show a larger magnetocaloric effect (MCE) than those showing a second-order magnetic transition. In second order magnetic phase transitions, the existence of short-range order and spin fluctuations above the Curie temperature ($T_{C}$) brings about a reduction in the maximum possible $\left|{\left(\frac{\partial M}{\partial T}\right)}_{B}\right|$ value, and the maximum MCE is thus reduced accordingly. In contrast, a first-order magnetic phase transition ideally occurs at a certain temperature (the transition temperature, $T_{t}$) and then the $\left|{\left(\frac{\partial M}{\partial T}\right)}_{B}\right|$ value should be theoretically infinitely large. Until now, the reported materials with a large MCE near room temperature are: Gd${}_{5}$(Si,Ge)${}_{4}$ [7]; Mn(As,Sb) [8,9]; (Mn,Fe)${}_{2}$(P,X) with X = As, Ge, Si [10,11,12]; LaFe${}_{13-x}$Si${}_{x}$ and its hydrides [13,14,15]; (Mn,Fe)${}_{2}$(P,Si,B) [16]; (Mn,Fe)${}_{2}$(P,Si,N) [17], NiMn-based Heusler alloys [18], FeRh [19]; MnCoGeB${}_{x}$ [20]; MnCoGe${}_{1-x}$Ga${}_{x}$ [21]; and MnCo${}_{1-x}$Fe${}_{x}$Si [22]. Among all above candidates for solid-state refrigerants, the (Mn,Fe)${}_{2}$(P,Si)-based materials are some of the most promising because they provide optimal conditions for practical applications (large MCE, low cost starting materials, and environmental benefits). (Mn,Fe)${}_{2}$(P,Si)-based materials crystallize in the hexagonal Fe${}_{2}$P-type structure (space group P-62m). In this structure, there are two specific metallic and non-metallic sites. For 3*d* transition metals, Mn preferentially occupies the 3*g* site at the pyramidal (x${}_{2}$, 0, 1/2) position, while Fe preferentially occupies the 3*f* site at the tetrahedral (x${}_{1}$, 0, 0) position. The non-metal elements P and Si occupy the 1*b* site at the (0, 0, 1/2) position and the 2*c* site at the (1/3, 1/3, 0) position with weakly preferred occupation of Si on the 2*c* site [23].

From an application point of view, (Mn,Fe)${}_{2}$(P,Si)-based materials need to have a very small hysteresis that should at least be smaller than their adiabatic temperature change ($\Delta T_{ad}$) and have a continuously tunable $T_{C}$ close to the working temperature, so that they can be used as a feasible magnetic refrigerant material. Since the discovery of the (Mn,Fe)${}_{2}$(P,Si) system, much effort has been put into tuning the Curie temperature ($T_{C}$) and reducing the thermal hysteresis ($\Delta T_{hys}$) without losing the giant MCE by varying the Mn/Fe and/or P/Si ratio [24], by substituting Mn and Fe by other transition metal and rare earths [25] or by substituting P or Si by B [26,27]. It has been found that boron substitution leads to an enhanced mechanical stability and a significant decrease in thermal hysteresis without losing the giant MCE [27,28]. The substitution of either P or Si by B leads to a strong increase in $T_{C}$, which complicates tuning $T_{C}$ by varying the boron content. In principle, one can keep the boron content constant and vary the Mn/Fe and/or P/Si ratio to tune $T_{C}$. However, the adjustment of the Mn/Fe and/or P/Si ratio often leads to either a decrease in the magnetization or an increase in the $\Delta T_{hys}$ , which is undesired for magnetic refrigeration. Hence, tuning $T_{C}$, while maintaining a thermal hysteresis as small as possible, is an essential step to practical magnetic refrigeration applications. It has recently been reported that the Co (Ni) substitution for either Mn or Fe lowers the Curie temperature and potentially reduces the thermal hysteresis [25,29,30]. Thus, co-doping of Co (Ni) and B in the (Mn,Fe)${}_{2}$(P,Si) system is expected to combine the positive effect of B substitution on improving the mechanical stability and reducing the thermal hysteresis, while $T_{C}$ can be tuned more easily than for sole B doping.

In this work, we show that it is possible to reduce the thermal hysteresis and tune $T_{C}$, while keeping a large MCE and good mechanical stability in (Mn,Fe)${}_{2}$(P,Si) compounds by Co(Ni) and B co-doping.

## 2. Results and Discussion

### 2.1. Mn${}_{1.00}$Fe${}_{0.85}$Co${}_{0.10}$P${}_{0.55-z}$Si${}_{0.45}$B${}_{z}$ Compounds

**Figure 1:**
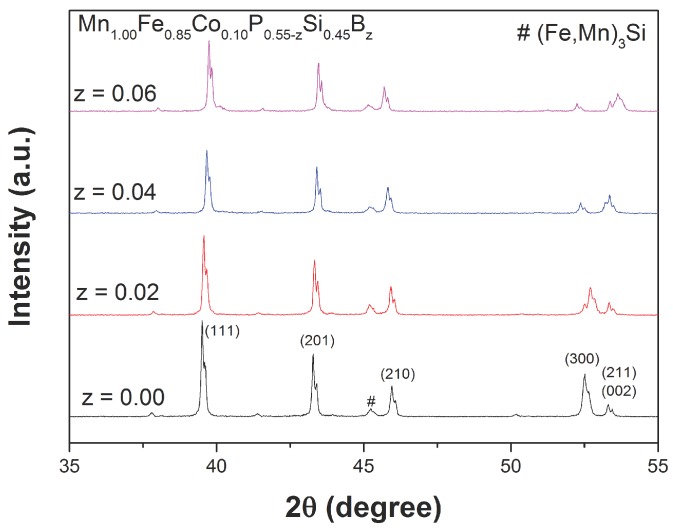
X-ray diffraction (XRD) patterns measured at 400 K (*T* > $T_{C}$) for the Mn${}_{1.00}$Fe${}_{0.85}$Co${}_{0.10}$P${}_{0.55-z}$Si${}_{0.45}$B${}_{z}$ compounds.

**Figure 2:**
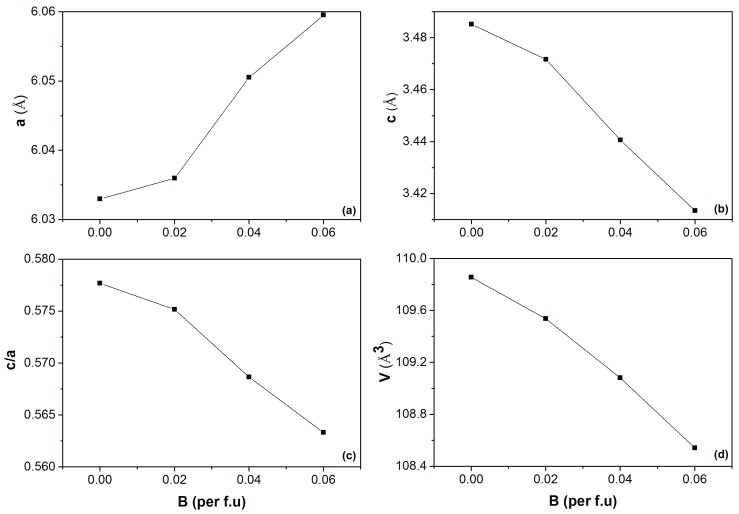
Lattice parameters *a* (**a**) and *c* (**b**), the c/a ratio (**c**) and the unit-cell volume *V* (**d**) obtained from XRD measurements at 400 K as a function of the boron content for the Mn${}_{1.00}$Fe${}_{0.85}$Co${}_{0.10}$P${}_{0.55-z}$Si${}_{0.45}$B${}_{z}$ compounds.

The influence of Co and B co-doping on the (Mn,Fe)${}_{2}$(P,Si)-based materials was first investigated in a batch of samples with a fixed Co concentration. Figure 1 shows the X-ray diffraction (XRD) patterns measured at 400 K (a temperature at which all the compounds are in the paramagnetic state) of Mn${}_{1.00}$Fe${}_{0.85}$Co${}_{0.10}$P${}_{0.55-z}$Si${}_{0.45}$B${}_{z}$ compounds, with a nominal composition of *z* = 0.00, 0.02, 0.04 and 0.06. All samples were found to crystallize in the hexagonal Fe${}_{2}$P-type structure (space group P-62m), indicating that the Co and B co-doping do not affect the Fe${}_{2}$P phase formation. A small amount of (Mn,Fe)${}_{3}$Si impurity phases (less than 5%), as often observed in this material system, is detected. The unit-cell volume decreases linearly for increasing B concentrations (about −0.23 Å${}^{3}$/at. % B), which is in good agreement with the results reported by Guillou et al. [27] for the (Mn,Fe)${}_{2}$(P,Si,B) system. Similar to the (Mn,Fe)${}_{2}$(P,Si,B) system, the lattice parameter *a* decreases, while the lattice parameter *c* increases, leading to a decrease in the c/a ratio with increasing B content, as shown in Figure 2.

**Figure 3:**
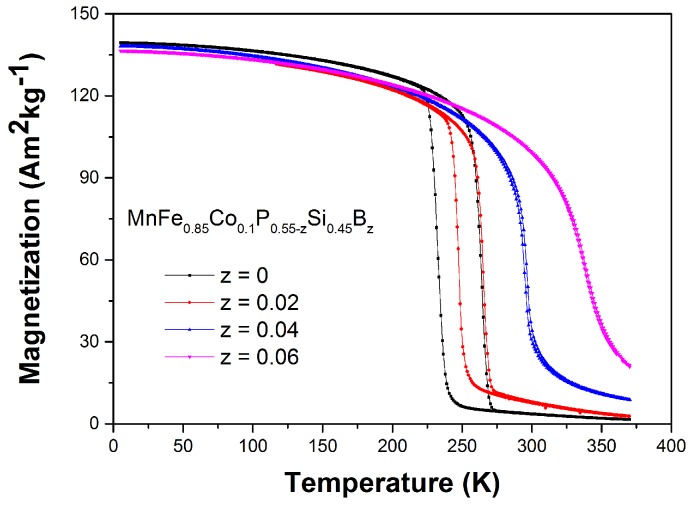
Magnetization as a function of temperature measured on heating and cooling in a magnetic field of 1 T for the Mn${}_{1.00}$Fe${}_{0.85}$Co${}_{0.10}$P${}_{0.55-z}$Si${}_{0.45}$B${}_{z}$ compounds. The temperature sweep rate is 2 K/min.

The temperature dependence of the magnetization (M−T curve) measured in a magnetic field of 1 T for the Mn${}_{1.00}$Fe${}_{0.85}$Co${}_{0.10}$P${}_{0.55-z}$Si${}_{0.45}$B${}_{z}$ series is shown in Figure 3. It is found that the Curie temperature ($T_{C}$) of the Mn${}_{1.00}$Fe${}_{0.85}$Co${}_{0.10}$P${}_{0.55-z}$Si${}_{0.45}$B${}_{z}$ compounds increases rapidly for increasing B concentrations, which is consistent with the evolution of the c/a ratio. Strikingly, there is a significant decrease in the thermal hysteresis when *z* increases. For the *z* = 0.00 and 0.02 samples, the magnetic transitions display a large thermal hysteresis, which is a clear signal for a first-order magnetic transition. Nevertheless, for the *z* = 0.04 and 0.06 samples, the magnetic transitions are close to the border between a first-order and a second-order magnetic transition, which is supported by a broad transition and a very small (or even not experimentally observable) thermal hysteresis. The corresponding values of the thermal hysteresis for *z* = 0.00, 0.02, 0.04 and 0.06 are $\Delta T_{hys}$ = 30.0, 17.0, 1.5, and 0.0 K, respectively. The average decrease in thermal hysteresis by B substitution is about 7 K/at. % B.

**Figure 4:**
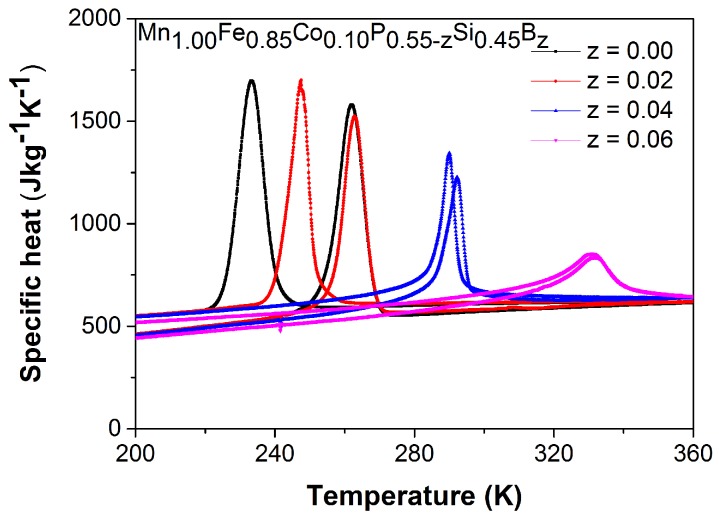
Specific heat derived from Differential scanning calorimetry (DSC) measurements for the Mn${}_{1.00}$Fe${}_{0.85}$Co${}_{0.10}$P ${}_{0.55-z}$Si${}_{0.45}$B${}_{z}$ compounds measured in the zero field upon cooling and heating.

As can be seen in Figure 4, the lower and broader peak in the specific heat curves at $T_{C}$ indicate that the magnetic transition of the Mn${}_{1.00}$Fe${}_{0.85}$Co${}_{0.10}$P${}_{0.55-z}$Si${}_{0.45}$B${}_{z}$ compounds changes gradually from a first-order to a second-order magnetic transition for increasing *z*. Moreover, there is a decrease in the latent heat as a function of the boron content. The corresponding latent heat values obtained by the integration of the curves in a zero field for *z* = 0.00, 0.02, 0.04 and 0.06 are 10.1, 8.0, 5.4 and 3.5 kJkg${}^{-1}$, respectively. From the above behavior, it is clear that an increase in the boron content weakens the first-order magnetic transition.

**Figure 5:**
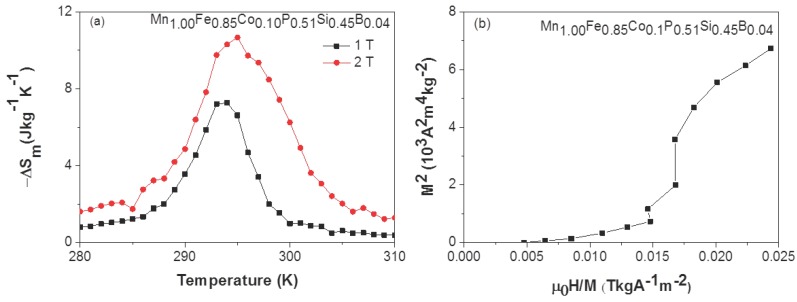
(**a**) Magnetic entropy change ($\Delta S_{m}$) as a function of temperature for a field change of 1 T (**black** markers) and 2 T (**red** markers); (**b**) Arrot plots derived from isofield $M_{B}$(T) curves measured upon cooling in the vicinity of $T_{C}$ for the Mn${}_{1.00}$Fe${}_{0.85}$Co${}_{0.10}$P${}_{0.51}$Si${}_{0.45}$B${}_{0.04}$ compound.

To evaluate the MCE of the Mn${}_{1.00}$Fe${}_{0.85}$Co${}_{0.10}$P${}_{0.51}$Si${}_{0.45}$B${}_{0.04}$ compound, the isofield magnetization $M_{B}$(T) curves are measured in the vicinity of $T_{C}$. The $M_{B}$(T) curves have been used (instead of the isothermal magnetization $M_{T}$(B) curves) to calculate the isothermal magnetic entropy change ($\Delta S_{m}$) because the application of the Maxwell equation on the $M_{B}$(T) curves is expected to prevent the so-called “spike” caused by a phase co-existence [31,32]. The isofield $M_{B}$(T) curves are first measured in the field upon cooling and then upon heating with a rate of 2 Kmin${}^{-1}$. For the calculation of $\Delta S_{m}$, only the data recorded upon cooling are used. Figure 5a shows $\Delta S_{m}$ as a function of temperature in a field change of 1 and 2 T for the Mn${}_{1.00}$Fe${}_{0.85}$Co${}_{0.10}$P${}_{0.51}$Si${}_{0.45}$B${}_{0.04}$ compound. The absolute values of $\Delta S_{m}$ are 7.3 and 10.7 Jkg${}^{-1}$K${}^{-1}$ for a field change of 1 and 2 T, respectively. The low value of the latent heat positively contributes to the large field dependence of the Curie temperature of $\frac{dT_{C}}{dB}=5.18$ KT${}^{-1}$ found in the Mn${}_{1.00}$Fe${}_{0.85}$Co${}_{0.10}$P${}_{0.51}$Si${}_{0.45}$B${}_{0.04}$ compound.

To obtain additional information on the nature of the phase transition, an Arrot plot (see Figure 5b) has been derived from the magnetic measurements in the vicinity of $T_{C}$. The S-shaped magnetization curve confirms the presence of a first-order magnetic transition for this sample.

**Figure 6:**
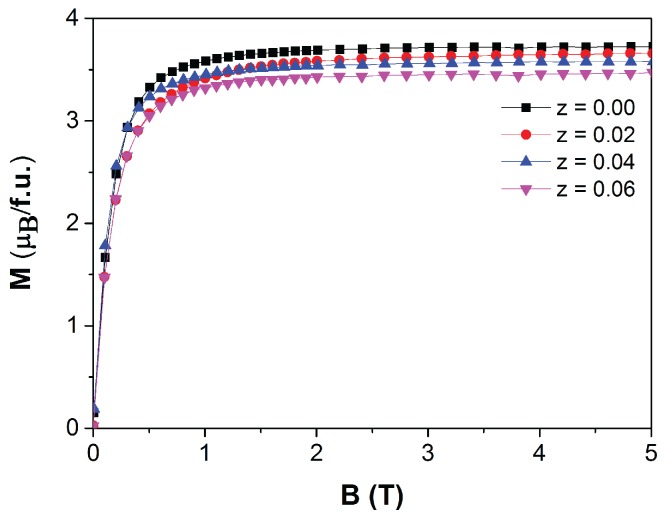
Field dependence of the magnetization of Mn${}_{1.00}$Fe${}_{0.85}$Co${}_{0.10}$P${}_{0.55-z}$Si${}_{0.45}$B${}_{z}$ compounds measured at a temperature of 5 K.

The magnetic field dependence of the magnetization of the Mn${}_{1.00}$Fe${}_{0.85}$Co${}_{0.10}$P${}_{0.55-z}$Si${}_{0.45}$B${}_{z}$ compounds with *z* = 0.00, 0.02, 0.04 and 0.06 at *T* = 5 K is shown in Figure 6. It is found that there is a slight decrease in the saturation magnetization ($M_{s}$) for increasing *z* (about −0.04 $\mu_{B}$/f.u. at. % B).

### 2.2. Mn${}_{1.00}$Fe${}_{0.95-z}$Co${}_{z}$P${}_{0.51}$Si${}_{0.45}$B${}_{0.04}$

The results in Section 2.1 indicate that the Mn${}_{1.00}$Fe${}_{0.85}$Co${}_{0.10}$P${}_{0.51}$Si${}_{0.45}$B${}_{0.04}$ compound, which shows a large isothermal entropy change and a small thermal hysteresis, is a very promising candidate for room-temperature magnetic refrigeration. Hence, a batch of samples based on a variation in the cobalt content Mn${}_{1.00}$Fe${}_{0.95-z}$Co${}_{z}$P${}_{0.51}$Si${}_{0.45}$B${}_{0.04}$ was prepared with the aim of tuning $T_{C}$ without losing the giant MCE or increasing $\Delta T_{hys}$ in the (Mn,Fe)${}_{2}$(P,Si) system.

**Figure 7:**
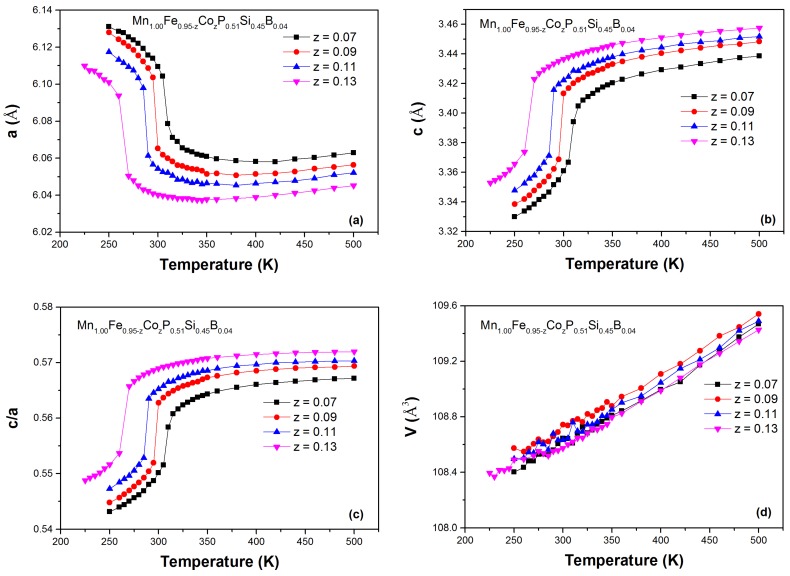
Temperature dependence of the lattice parameters *a* (**a**) and *c* (**b**), the c/a ratio (**c**) and the unit-cell volume *V* (**d**) for the Mn${}_{1.00}$Fe${}_{0.95-z}$Co${}_{z}$P${}_{0.51}$Si${}_{0.45}$B${}_{0.04}$ compounds with *z* = 0.07, 0.09, 0.11 and 0.13, derived from XRD patterns measured upon heating.

The evolution of the lattice parameters and unit-cell volume as a function of temperature for the Mn${}_{1.00}$Fe${}_{0.95-z}$Co${}_{z}$P${}_{0.51}$Si${}_{0.45}$B${}_{0.04}$ is presented in Figure 7. The most prominent feature is the abrupt jump in the lattice parameters at the ferro to paramagnetic phase transition. This confirms the existence of a first-order magneto-elastic transition (FOMET). Three main features can be noticed for the influence of Co and B co-doping. First, the lattice parameter *a* decreases, while *c* increases, both in the ferromagnetic (FM) state and in the paramagnetic (PM) state for an increasing Co content. Second, the combined evolution of *a* and *c* results in an increase in the c/a ratio, both in the FM state and in the PM state. Finally, there is a very small volume change at the magnetic transition for these samples because the *a* and *c* parameters change in the opposite direction. It is worth noting that, similar to the (Mn,Fe)${}_{2}$(P,Si,B) system, there is no noticeable ΔV at the FOMET ( ΔV/V < 0.05%). Guillou et al. [26] established that the absence of a unit-cell volume change at the transition improves the mechanical stability in the (Mn,Fe)${}_{2}$(P,Si,B) system in comparison to the (Mn,Fe)${}_{2}$(P,Si) compounds. The Co and B co-doping still takes advantage of the strong impact of the B substitution to provide an enhanced mechanical stability.

**Figure 8:**
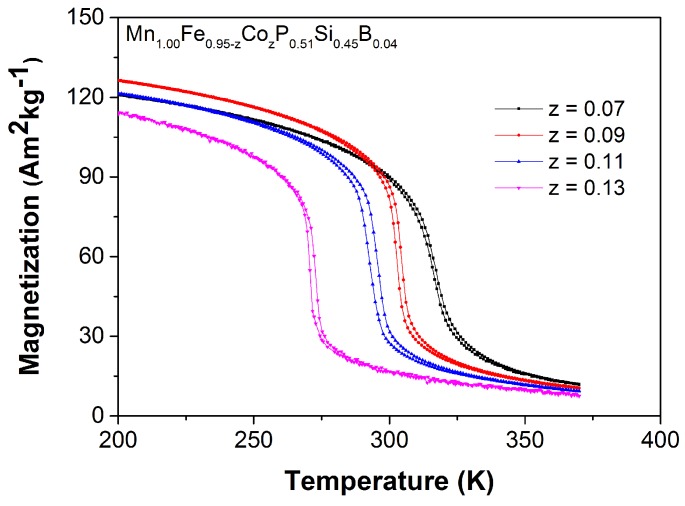
Magnetization as a function of temperature measured on heating and cooling in a magnetic field of 1 T for the Mn${}_{1.00}$Fe${}_{0.95-z}$Co${}_{z}$P${}_{0.51}$Si${}_{0.45}$B${}_{0.04}$ compounds. The temperature sweep rate is 2 K/min.

Figure 8 shows the M-T curves measured in a magnetic field of 1 T for the Mn${}_{1.00}$Fe${}_{0.95-z}$Co${}_{z}$P${}_{0.51}$Si${}_{0.45}$B${}_{0.04}$ series. Consistent with the results reported by Huliyageqi et al. [30], it is found that an increase in the Co concentration lowers the Curie temperature, while the $\Delta T_{hys}$ value is retained to be very small ($\Delta T_{hys}$ = 1–2 K) with a sharp transition at $T_{C}$. The corresponding values of $T_{C}$ obtained from the heating curves for *z* = 0.07, 0.09, 0.11 and 0.13 are 316, 304, 295 and 272 K, respectively. Hence, keeping the B content constant and varying the Co content provides a handle to tune $T_{C}$ in a broad range around room temperature, while maintaining a very small thermal hysteresis. The variations in $T_{C}$, $\Delta S_{m}$, $\Delta T_{hys}$ as a function of Co content for the Mn${}_{1.00}$Fe${}_{0.95-z}$Co${}_{z}$P${}_{0.51}$Si${}_{0.45}$B${}_{0.04}$ compounds are summarized in Table 1.

**Figure 9:**
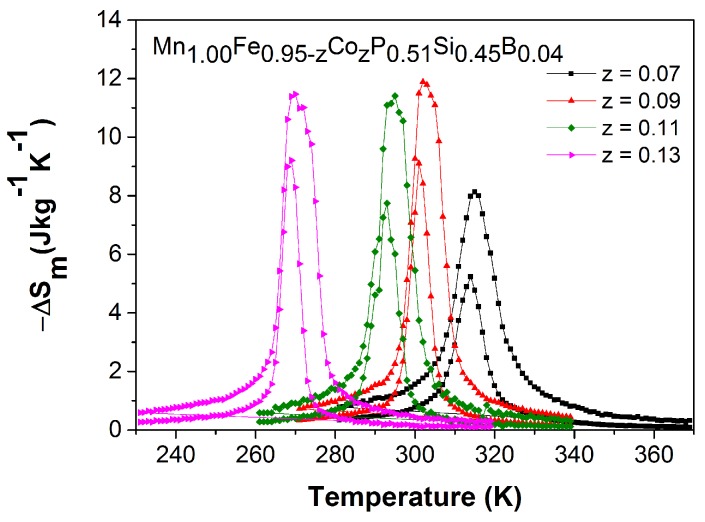
Magnetic entropy change as a function of temperature for a field change of 1 T (lower curve) and 2 T (upper curve) derived from isofield $M_{B}$(T) curves measured upon cooling in the vicinity of $T_{C}$ for the Mn${}_{1.00}$Fe${}_{0.95-z}$Co${}_{z}$P${}_{0.51}$Si${}_{0.45}$B${}_{0.04}$ compounds.

Although Co and B co-doping leads to a partial loss of magnetic transition sharpness compared to (Mn,Fe)${}_{2}$(P,Si)-based materials, the $\Delta S_{m}$ derived from the isofield magnetization curves, presented in Figure 9, is still comparable to those reported for giant-MCE materials like (Mn,Fe)${}_{2}$(P,Si,B) [26], Gd${}_{5}$Si${}_{2}$Ge${}_{2}$, Heusler alloys and La(Fe,Si)${}_{13}$H${}_{y}$ [6]. The peak values, which are weakly depending on the Co content, are in the range of 5–9 and 9–12 JK${}^{-1}$kg${}^{-1}$ for a field change of 1 and 2 T, respectively.

**Figure 10:**
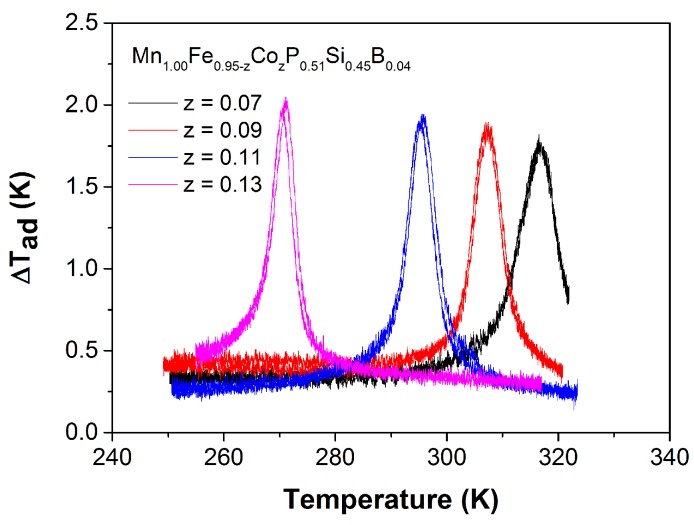
Temperature dependence of the adiabatic temperature change obtained by direct measurements for the Mn${}_{1.00}$Fe${}_{0.95-z}$Co${}_{z}$P${}_{0.51}$Si${}_{0.45}$B${}_{0.04}$ compounds in a magnetic field change of ΔB = 1.1 T.

The adiabatic temperature change obtained from the direct measurements on the Mn${}_{1.00}$Fe${}_{0.95-z}$Co${}_{z}$P${}_{0.51}$Si${}_{0.45}$B${}_{0.04}$ compounds is shown in Figure 10. For a field change of 1.1 T, the $\Delta T_{ad}$ of the Mn${}_{1.00}$Fe${}_{0.95-z}$Co${}_{z}$P${}_{0.51}$Si${}_{0.45}$B${}_{0.04}$ powder samples varies from 1.8 to 2.0 K, which is comparable or slightly higher than those of the (Mn,Fe)${}_{2}$(P,Si)-based materials [33]. It should be noted that we used powder samples rather than bulk samples for these direct measurements, which leads to a potential underestimation of the adiabatic temperature change due to lower thermal conductance between the sample and the thermocouple. In other words, the real values of Mn${}_{1.00}$Fe${}_{0.95-z}$Co${}_{z}$P${}_{0.51}$Si${}_{0.45}$B${}_{0.04}$ compounds should be higher. Interestingly, there is hardly any change in the saturation magnetization (M${}_{s}$) for increasing Co content. Therefore, when combining a fixed B concentration with varying the Co content, the size of the magnetic moments and the thermal hysteresis of (Mn,Fe,Co)${}_{2}$(P,Si,B)-based materials are retained, while keeping a large MCE in a wide range of working temperatures.

### 2.3. Mn${}_{1.00}$Fe${}_{0.95-z}$Ni${}_{z}$P${}_{0.51}$Si${}_{0.45}$B${}_{0.04}$

The experimental results in Section 2.2 show that co-doping of Co and B in the (Mn,Fe)${}_{2}$(P,Si) system offers a new control parameter to tune $T_{C}$ while keeping a small thermal hysteresis and preserving the positive effect of boron addition on the mechanical stability. However, Co is quite expensive, which affects fabrication costs, one of the most important factors for commercial applications. Hence, it is desirable to find another element that can replace Co, without any significant effect on both MCE and mechanical properties in (Mn,Fe,Co)${}_{2}$(P,Si,B) compounds, in order to lower fabrication costs. The experimental results from Wada et al. [25] show that the substitution of Fe by Ni in the (Mn,Fe)${}_{2}$(P,Si) system has the same effect as Co substitution on both the Curie temperature and the thermal hysteresis. Moreover, Ni is three times cheaper than Co [34]. This suggests that Ni is an ideal choice to replace Co in the (Mn,Fe,Co)${}_{2}$(P,Si,B) system.

**Figure 11:**
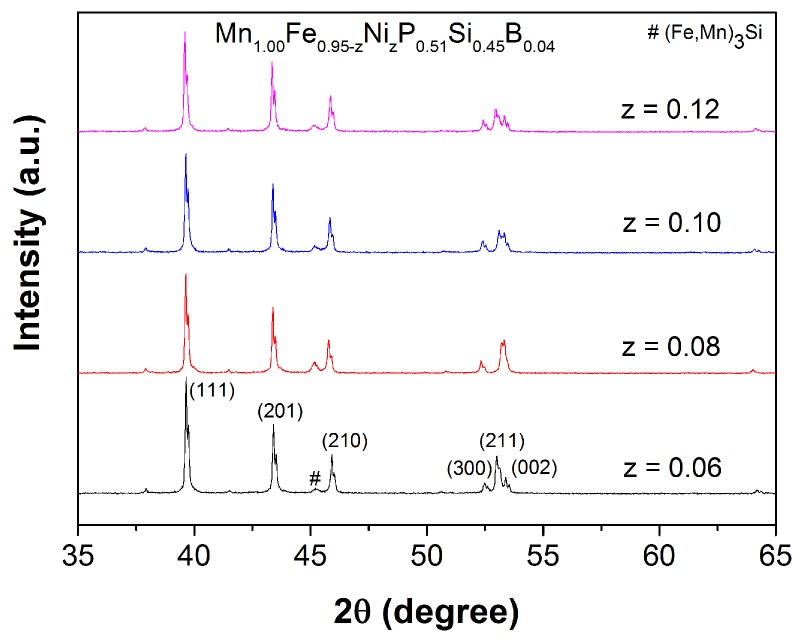
XRD patterns measured at 400 K for the Mn${}_{1.00}$Fe${}_{0.95-z}$Ni${}_{z}$P${}_{0.51}$Si${}_{0.45}$B${}_{0.04}$ compounds.

The XRD patterns of Mn${}_{1.00}$Fe${}_{0.95-z}$Ni${}_{z}$P${}_{0.51}$Si${}_{0.45}$B${}_{0.04}$ compounds with *z* = 0.06, 0.08, 0.10 and 0.12 shown in Figure 11 indicate that the co-substitution of Fe by Ni and P by B does not change the crystal structure. All of the samples crystallize in the hexagonal Fe${}_{2}$P-type structure (space group P-62m). The structure refinement results show that an increase in the Ni content leads to an increase in the c/a ratio resulting in a lower $T_{C}$.

**Figure 12:**
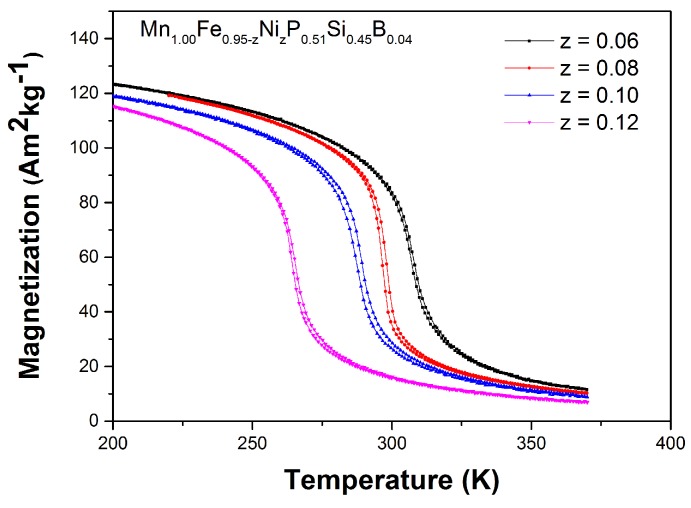
Magnetization as a function of temperature measured on heating and cooling in a magnetic field of 1 T for the Mn${}_{1.00}$Fe${}_{0.95-z}$Ni${}_{z}$P${}_{0.51}$Si${}_{0.45}$B${}_{0.04}$ compounds. The applied sweep rate is 2 K/min.

The magnetization versus temperature curves of the Mn${}_{1.00}$Fe${}_{0.95-z}$Ni${}_{z}$P${}_{0.51}$Si${}_{0.45}$B${}_{0.04}$ compounds upon cooling and heating in an applied field of 1 T shown in Figure 12 indicate that all of the samples have sharp first-order magnetic transitions around $T_{C}$. Similar to Co doping, an increase in Ni concentration leads to a decrease in $T_{C}$, which is consistent with the results reported by Wada et al. [25]. It is worth noting that the change in Ni content does not significantly affect the thermal hysteresis. While $T_{C}$ amounts to 308, 298, 289 and 265 K for the samples with *z* = 0.06, 0.08, 0.10 and 0.12, respectively, the thermal hysteresis remains constant at 1–2 K.

**Figure 13:**
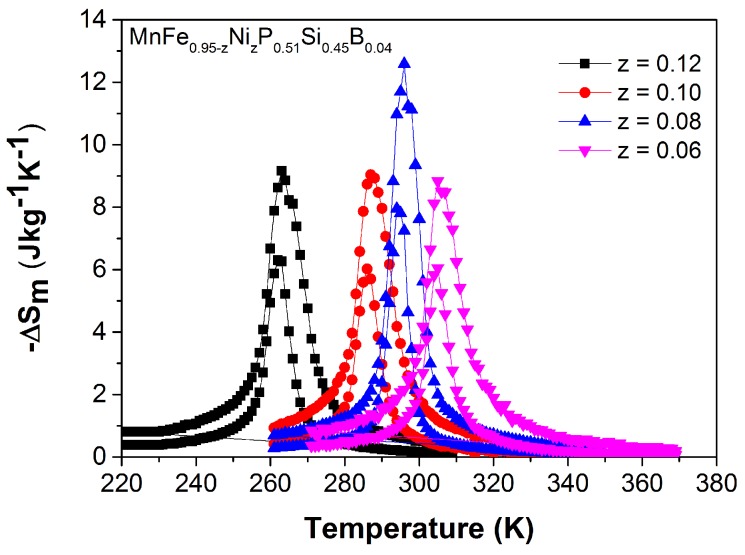
Magnetic entropy change as a function of temperature for a field change of 1 T (lower curve) and 2 T (upper curve) derived from isofield $M_{B}$(T) curves measured upon cooling in the vicinity of TC for the Mn${}_{1.00}$Fe${}_{0.95-z}$Ni${}_{z}$P${}_{0.51}$Si${}_{0.45}$B${}_{0.04}$ compounds.

Figure 13 shows $\Delta S_{m}$ of the Mn${}_{1.00}$Fe${}_{0.95-z}$Ni${}_{z}$P${}_{0.51}$Si${}_{0.45}$B${}_{0.04}$ compounds in a field change of 1 and 2 T. The $\Delta S_{m}$ was derived from the isofield magnetization curves using the Maxwell relation. The peak values, which are weakly depending on the Ni content, are in the range of 6–8 and 9–13 JK${}^{-1}$kg${}^{-1}$ for a field change of 1 and 2 T, respectively. Compared to the Co and B co-doping system, the isothermal magnetic entropy change of Ni and B co-doping system is slightly lower.

**Figure 14:**
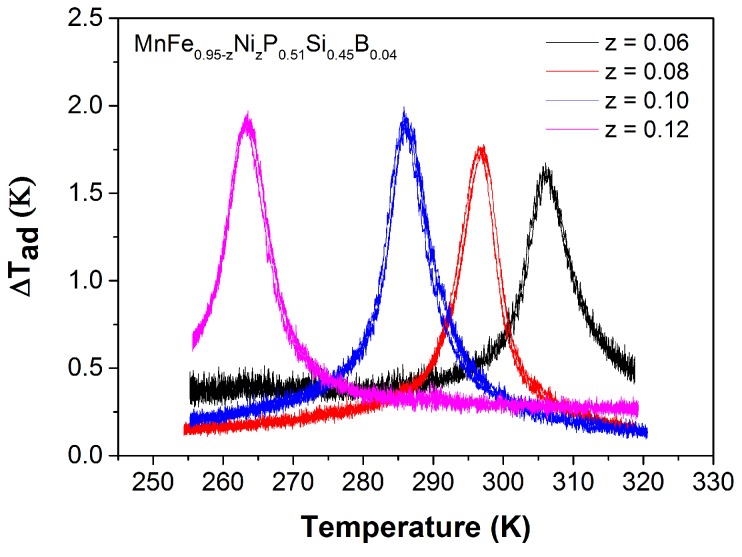
Temperature dependence of the adiabatic temperature change obtained by direct measurements for a magnetic field change of ΔB = 1.1 T.

The adiabatic temperature change $\Delta T_{ad}$ derived from the direct measurements is shown in Figure 14. For a field change of 1.1 T, the $\Delta T_{ad}$ varies from 1.7 to 1.9 K. The $\Delta T_{ad}$(T) of Mn${}_{1.00}$Fe${}_{0.95-z}$Ni${}_{z}$P${}_{0.51}$Si${}_{0.45}$B${}_{0.04}$ powder is comparable or slightly lower than that of Co and B co-doping samples. Hence, along with Co and B co doping, co-doping of Ni and B also offers an additional control parameter to tune $T_{C}$ and adjust the thermal hysteresis while maintaining a large MCE and improving mechanical properties in (Mn,Fe)${}_{2}$(P,Si) compounds. This makes the Mn${}_{1.00}$Fe${}_{0.95-z}$Ni${}_{z}$P${}_{0.51}$Si${}_{0.45}$B${}_{0.04}$ compounds also very promising for room-temperature magnetic refrigeration.

## 3. Materials and Methods

Three series of samples were prepared in the same way: high-energy ball milling first followed by solid-state sintering. In the first series, a variation of the boron content was applied for Mn${}_{1.00}$Fe${}_{0.85}$Co${}_{0.10}$P${}_{0.55-z}$Si${}_{0.45}$B${}_{z}$. In the second series, a variation of the cobalt content was applied for Mn${}_{1.00}$Fe${}_{0.95-z}$Co${}_{z}$P${}_{0.51}$Si${}_{0.45}$B${}_{0.04}$. In the last series, a variation of the nickel content was applied for Mn${}_{1.00}$Fe${}_{0.95-z}$Ni${}_{z}$P${}_{0.51}$Si${}_{0.45}$B${}_{0.04}$. Stoichiometric quantities of the starting materials Mn, Fe, Co, red P, B and Si powders were ground in a planetary ball mill for 10 h with a constant rotation speed of 380 rpm. The planetary ball mill Fritsch Pulverisette (Fritsch International, Rudolstadt, Germany) with the grinding jars and ball made of tungsten carbide (7 balls with a diameter of 10 mm per jar) has been used to prepared all samples. The milled powders were compacted into small tablets (with a diameter of 12 mm and a height of 5–10 mm) with a pressure of 150 kgfcm${}^{-2}$. After pressing, the tablets were sealed in quartz ampoules under 200 mbar of Ar before employing the double-step sintering described in Ref. [35] and quenching into water. It is worth noting that all samples have good mechanical stability, which was supported by the absence of cracking after cooling the samples in liquid nitrogen.

The XRD data of all samples were collected at various temperatures in a PANalytical X-pert Pro diffractometer (Panalytical, Almelo, The Netherlands) equipped with an Anton Paar TTK450 low-temperature chamber (Panalytical, Almelo, The Netherlands) using Cu-K${}_{\alpha }$ radiation and were refined using the Fullprof program [36]. A differential scanning calorimeter (DSC) equipped with a liquid nitrogen cooling system was used to measure the specific heat. Magnetic measurements were carried out in a Superconducting Quantum Interference Device (SQUID) magnetometer (MPMS XL, Quantum Design International, San Diego, CA, USA).

Direct measurements of the adiabatic temperature change $\Delta T_{ad}$ for powder samples were performed in a home-built experimental setup, which is designed to track the temperature of the magnetocaloric materials during magnetization and demagnetization processes while the surrounding temperature is slowly scanned over the temperature range of interest. For the direct measurements, a thermocouple was put in the middle of the sample holder, which is a small pylon-shaped plastic cup. Then, the sample holder was filled with the sample. Kapok was put on top of the powder to compress the powder, which helps increase the heat contact of the sample with the thermocouple. Finally, the sample holder was covered by a plastic cap. During the measurements, the sample holders moved in and out a magnetic field generated by two permanent magnets at a frequency of 0.1 Hz. The temperature sweep rate of a climate chamber, which regulated the surrounding temperature, was about 0.5–1.5 K/min. This is relatively low with respect to the dT/dt related to the response time of the thermocouple (about 150 K/min). Hence, this set-up can be considered as operating under quasi-adiabatic conditions [37].

To ensure the reproducibility of the measurements, the measurements were carried out upon warming and cooling three times. Only the last warming and cooling $\Delta T_{ad}$(T) curves are presented in this work.

## 4. Conclusions

(Mn,Fe,Co)${}_{2}$(P,Si,B)-based and (Mn,Fe,Ni)${}_{2}$(P,Si,B)-based materials were prepared by high-energy ball milling and solid-state reaction. The effect of the co-substitution of Fe by Co or Ni and P by B on $T_{C}$, $\Delta T_{hys}$ and the MCE has been studied systematically by XRD, DSC, and magnetic and direct temperature change measurements. The experimental results show that, by Co (Ni) and B co-doping, the thermal hysteresis is tunable to very small values (or even not experimentally observable) while maintaining a large MCE in a wide temperature range around room temperature. $T_{C}$ can be tuned from 272 to 316 K and from 265 to 308 K by varying Co content and Ni content, respectively. Therefore, co-substitution of Fe by Co (Ni) and P by B is found to be a promising approach to tune the Curie temperature, while keeping the thermal hysteresis as small as possible, maintaining a giant MCE and improving the mechanical stability in the (MnFe)${}_{2}$(P,Si) system. This makes (Mn,Fe,Co)${}_{2}$(P,Si,B) and (Mn,Fe,Ni)${}_{2}$(P,Si,B) compounds highly promising for near room-temperature magnetic refrigeration. In other words, Co-B and Ni-B co-doping offers new control parameters to bring practical magnetic cooling near room temperature a step closer.

## Figures and Tables

**Table 1:** Curie temperature ($T_{C}$) derived from the magnetization curves measured on heating, the isothermal entropy change ($\Delta S_{m}$) derived from the isofield magnetization curves in a field change of 0.5, 1.0, 1.5 and 2.0 T, thermal hysteresis ($\Delta T_{hys}$) derived from the magnetization curves measured in 1 T upon cooling and heating for the Mn${}_{1.00}$Fe${}_{0.95-z}$Co${}_{z}$P${}_{0.51}$Si${}_{0.45}$B${}_{0.04}$ compounds.

*z*	$T_{C}$	$\Delta S_{m}$ (JK${}^{-1}$kg${}^{-1}$)				$\Delta T_{\mathrm{hys}}$ (K)
		ΔB = 0.5 T	ΔB = 1.0 T	ΔB = 1.5 T	ΔB = 2.0 T	
0.07	316	2.7	5.3	6.8	8.1	1.3
0.09	304	5.0	9.1	10.7	11.9	1.7
0.11	295	3.7	7.7	10.0	11.4	2.5
0.13	272	7.7	9.2	10.6	11.5	1.9

## Abbreviations

The following abbreviations were used in this manuscript:

MCE	Magnetocaloric effect
$\Delta S_{m}$	Isothermal magnetic entropy change
$\Delta T_{ad}$	Adiabatic temperature change
FOMT	First-order magnetic transition
$T_{C}$	Curie temperature
XRD	X-ray diffraction
DSC	Differential scanning calorimeter
SQUID	Superconducting quantum interference device
$\Delta T_{hys}$	Thermal hysteresis
FOMET	First-order magneto-elastic transition
FM	Ferromagnetic
PM	Paramagnetic
